# Radiotherapy improves the survival of patients with stage IV NSCLC: A propensity score matched analysis of the SEER database

**DOI:** 10.1002/cam4.1776

**Published:** 2018-09-21

**Authors:** Rui Zhang, Ping Li, Qin Li, Yunfeng Qiao, Tangpeng Xu, Peng Ruan, Qibin Song, Zhenming Fu

**Affiliations:** ^1^ Cancer Center Renmin Hospital of Wuhan University Wuhan China

## Abstract

**Objectives:**

The survival advantage of radiotherapy (RT) for patients with stage IV non‐small cell lung cancer (NSCLC) has not been adequately evaluated.

**Methods:**

We analyzed stage IV NSCLC patients enrolled from the Surveillance, Epidemiology, and End Results (SEER) registry through January 2010 to December 2012. Propensity score (PS) analysis with 1:1 nearest neighbor matching method was used to ensure well‐balanced characteristics of all comparison groups by histological types and metastatic sites. Kaplan‐Meier and Cox proportional hazardous model were used to evaluate the overall survival (OS), cancer‐specific survival (CSS), and corresponding 95% confidence interval (95%CI).

**Results:**

Generally speaking, there was a trend toward improved OS and CSS for using RT to stage IV NSCLC patients for any metastatic sites and for any histological types except adenocarcinoma (AD). Radiotherapy significantly improved the survival of NSCLC patients with metastasis to brain (*P* < 0.001), especially for AD (*P* < 0.001). For stage IV lung cancer patients with squamous cell carcinoma (SQC), RT for any metastatic sites could universally improve the OS (*P* < 0.001) and CSS (*P* < 0.001). In particular, RT was also associated with improving OS (*P* < 0.001) and CSS (*P* = 0.012) for stage IV patients with metastases of two or more sites, ie, polymetastatic disease. Furthermore, for those stage IV SQC patients without metastasis, RT, most likely to the primary site, also significantly improved the survival (*P* < 0.001).

**Conclusions:**

The results support that RT might improve the survival of patients with metastatic NSCLC in a PS‐matched patient cohort from the large SEER database. It is prudent to carefully select patients for RT in metastatic NSCLC.

## 1 OBJECTIVES

Past treatment for patients with stage IV non‐small cell lung cancer (NSCLC) has mainly been platinum‐based chemotherapy, which corresponds to a median overall survival (OS) of 8‐10 months.^1^ In 2003, the U.S. Food and Drug Administration (FDA) approved gefitinib (Iressa^®^), an epidermal growth factor receptor (EGFR) tyrosine kinase inhibitor (TKI), for the treatment of patients with locally advanced or metastatic NSCLC after failure of chemotherapy.^2^ Since then, an array of this kind of molecular targeted therapy^3^ and immunotherapy^4^ has been approved by the FDA for the treatment of metastatic NSCLC, especially for adenocarcinoma (AD). With the development of these new treatment options, the efficacy of systemic therapy in metastatic NSCLC has been continuously, although slowly, improving.^5^ With the prolonged survival of metastatic NSCLC patients, the importance of local control of primary and metastatic lesions is emerging. Thus, several strategies involving systemic therapy paired with a local treatment modality, such as radiotherapy (RT), have been explored.

It has been shown that using RT to control primary and metastatic tumors can reduce intra‐thoracic disease burden, bronchial/vascular compression, and pulmonary symptoms.^6^ In addition, local control of primary and metastatic tumors might result in better OS.^7^, ^8^, ^9^ Recently, a phase III randomized controlled trial (RCT) showed that thoracic radiotherapy (TRT) also improved OS of patients with extensive‐stage small cell lung cancer (SCLC) who responded to chemotherapy.^10^ Moreover, it has been reported that RT of the primary tumor may confer a survival benefit for certain patients with stage IV NSCLC.^11^, ^12^ Studies have also shown that RT of metastatic sites for either oligometastasis^13^ or non‐oligometastasis, that is, polymetastasis,^14^ may significantly improve the survival of stage IV NSCLC patients. Furthermore, advancements in RT techniques may reduce its toxicity to normal tissue and allow increased tumor doses, which may further improve the efficacy.^15^ Nevertheless, limited prospective RCT data are available to define the roles of RT in stage IV NSCLC. Whether or not RT in addition to chemotherapy is beneficial for OS in patients with metastatic NSCLC has not been adequately studied.

Therefore, we analyzed a large database from the Surveillance, Epidemiology, and End Results (SEER) registry representative of the entire U.S. patient population through conventional and propensity score matching (PSM) approaches.

## 2 MATERIALS AND METHODS

### 2.1 Participants and methods

#### 2.1.1 Study population and data sources

**Figure 1:**
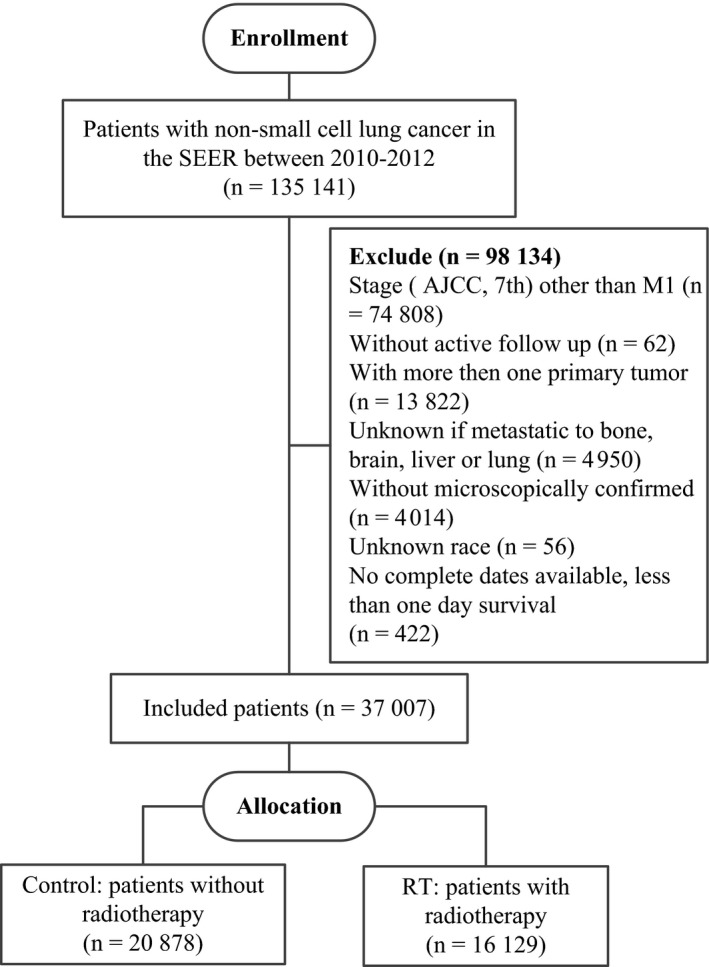
The flowchart of study population selection

The SEER database encompasses population‐based cancer registries that cover approximately 28% of the U.S. population and includes basic demographics and some clinical characteristics.^16^ Eligible participants who were diagnosed as lung and bronchus cancer cases with a pathological report of AD, squamous cell carcinoma (SQC), large cell carcinoma (LCC), or any other specified or unspecified carcinoma from January 2010 to December 2012 were identified from the SEER database. The NSCLC categorization methods were described previously by Lewis et al^17^ SEER*stat software (version 8.3.4, NIH, USA) was used to select patients. Inclusion criteria for this study were as follows: an M1 stage tumor according to the American Joint Committee on Cancer (*AJCC*) 7th edition; only one primary tumor; complete data on age, race, gender, tumor size, radiation recode, and metastases of the bone, liver, brain, lung at diagnosis; active follow‐up; and more than 0 days of survival. A total of 37 007 patients were recruited in this study, including both those who received RT (cases, n = 16 129) and those who did not (controls, n = 20 878; Figure 1).

#### 2.1.2 Propensity score matching

In an observational study, some selection bias always exists, which causes an imbalanced distribution of the confounding factors between two groups. A propensity score (PS) is the conditional probability of assignment to a particular treatment given a vector of observed covariates.^18^ To reduce selection bias and imbalanced distributions of the confounding factors, the PSM method was used in this study.^19^ Parameters that were significant in the univariate logistic regression models were entered into a multivariate logistic regression model to calculate PSs for each patient in the RT group and the non‐RT group. The PSM plug‐in^20^ of SPSS software was used to estimate the PS of each case. Then, PSM was performed using 1:1 nearest neighbor matching with a caliper of 0.02 to accept a matched pair. Chi‐squared tests were used to examine the covariate balance before and after PSM. In this study, PSM was performed between the RT group and the non‐RT group of each subgroup.

### 2.2 Statistical analysis

General linear models or Mantel‐Haenszel chi‐squared tests were used to compare the distribution of demographic characteristics. The Kaplan‐Meier method was used to analyze the primary outcomes of OS and cancer‐specific survival (CSS). A multivariate Cox proportional hazards model was performed to evaluate the hazard ratio (HR) and 95% confidence interval (CI). Variables selected for multivariate analysis included age (≤65 years or >65 years^21^), sex, race (white, black, or other), marital status (married or unmarried), histology (ADC, SQC, LCC, or other specified or unspecified carcinoma), grade (1, 2, 3, 4, or unknown), sites of distant metastasis (brain, bone, liver and lung), number of distant metastatic sites (number of distant metastatic sites in the brain, bone, liver and lung), chemotherapy (yes or no), surgery (yes or no), and RT (yes or no). *P*‐values for linear trends were derived from regression models treating target categories (excluding unknowns) as continuous covariates. *P*‐values ≤0.05 (2‐sided) were considered statistically significant. All analyses were conducted using SPSS 23 (IBM Corp, Armonk, NY, USA).

## 3 RESULTS

A total of 37 007 cases were included in this study. The distributions of the patient characteristics for both study groups, that is, cases and controls, are presented in Table 1. PSM included the variables of age, gender, race, marital status, histological type, grade, T stage, N stage, chemotherapy, surgery, RT, and metastatic sites of bone, brain, liver, and lung. Nearly half of the patients received RT. Before PSM, cases were more likely than controls to be younger and to have well‐defined tumor sizes and metastasis to bone or brain. Although significant differences were still observed in some variables, the distributions of most demographic and clinical factors were well balanced between the cases and controls after PSM.

**Table 1:** Selected baseline characteristics for study population by study groups, the Surveillance, Epidemiology, and End Results (SEER) database, 2010‐2012

Characteristic	Before PSM			After PSM^a^		
	No RT (n* *=* *20 878)	RT (n* *=* *16 129)	*P* b	No RT (n* *=* *10 583)	RT (n* *=* *10 583)	*P* b
Age [Years, mean (SD)]	68.9 (11.2)	64.7 (10.9)	<0.001	66.7 (11.1)	66.4 (11.2)	0.042
Sex (Female, %)	44.9	43.8	0.043	43.2	44.0	0.280
Race (White, %)	77.7	78.4	0.108	78.3	76.6	0.005
Marital (Married, %)	47.7	52.3	<0.001	49.4	49.5	0.901
Histology (%)						
AD	58.4	55.0	<0.001	56.1	55.9	0.206
SQC	18.1	21.2		19.8	20.6	
Other	4.9	4.4		4.6	4.5	
LCC	2.3	2.5		2.4	2.5	
Unspecified	16.3	16.8		17.1	16.5	
Grade (%)						
1	2.7	1.8	<0.001	1.9	2.0	0.305
2	10.3	11.2		11.6	11.3	
3	24.7	29.2		28.2	29.2	
4	1.6	1.7		1.6	1.8	
Unknown	60.8	56.1		56.7	55.6	
T (%)						
T0‐T2	31.0	35.2	<0.001	33.5	34.3	0.119
T3‐T4	53.0	55.0		55.9	54.6	
Unknown	16.0	9.8		10.6	11.2	
N (%)						
N0	26.2	21.2	<0.001	21.6	22.1	0.098
N1	7.2	8.3		8.0	8.3	
N2	41.3	46.7		45.4	43.9	
N3	17.5	19.4		20.1	20.1	
Unknown	7.8	4.4		4.9	5.5	
Number of metastatic sites of lung, brain, bone and liver (%)^c^						
0	32.8	16.2	<0.001	25.7	24.1	0.029
1	46.0	49.7		46.6	46.8	
2	15.9	23.9		20.4	21.4	
3	4.6	8.3		6.0	6.4	
4	0.7	1.8		1.4	1.2	
Bone metastasis (Yes, %)^d^	30.1	44.7	<0.001	44.3	43.9	0.472
Brain metastasis (Yes, %)^d^	10.9	44.4	<0.001	21.2	23.6	<0.001
Liver metastasis (Yes, %)^d^	20.1	15.3	<0.001	16.8	17.9	0.034
Lung metastasis (Yes, %)^d^	33.3	25.5	<0.001	28.4	28.7	0.616
Chemotherapy (Yes, %)	56.2	37.8	<0.001	56.7	54.9	0.010
Surgery (Yes, %)	3.8	3.2	0.002	3.2	3.3	0.462

n, number of cases/controls; PSM, propensity score matching; RT, radiotherapy; AD, adenocarcinoma; SQC, squamous cell carcinoma; Other, other specified carcinoma; LCC, large cell carcinoma; Unspecified, unspecified malignant neoplasm.

a PSM were done using variables including: age, gender, race, marital, histological types, grade, T stage, N stage, chemotherapy, surgery, radiotherapy, and metastatic sites of bone, brain, liver, and lung.

b Derived from ANOVA for continuous variables and *X* ^2^ test for categorical variables.

c Number of distant metastasis include four sites of distant metastasis at diagnose SEER provided: bone; brain; liver; lung.

d Bone, brain, liver, and lung are four sites of distant metastasis at diagnose SEER provided.

All the baseline characteristics and selected variables were included in a univariate analysis between the cases and controls in relation to both OS and CSS. Table 2 shows all the significant variables in the univariate analysis in addition to the results when all the variables were entered into a multivariate model. As expected, older age, male, higher histology grade and T stage, larger tumor sizes, and more distant metastasis were associated with poorer OS and CSS. Receiving chemotherapy, surgery, and RT were all strongly associated with better survival (*P *<* *0.001). Histology of AD was also found to be associated with better survival. However, white, unmarried patients were found to be associated with slight but significantly worse survival, which was possibly partially due to the large sample size.

**Table 2:** Univariate and multivariate analyses of OS and CSS for different variables of NSCLC before PSM

Characteristic^b^	Univariate				Multivariate			
	OS		CSS		OS		CSS	
	HR (95% CI)^a^	*P* a	HR (95%CI)^a^	*P* a	HR (95%CI)^a^	*P* a	HR (95%CI)^a^	*P* a
Age (<66 as ref.)	1.3 (1.3‐1.31)	<0.001	1.3 (1.2‐1.3)	<0.001	1.2 (1.1‐1.2)	<0.001	1.1 (1.1‐1.2)	<0.001
Gender (Female as ref.)	1.2 (1.2‐1.3)	<0.001	1.2 (1.2‐1.2)	<0.001	1.2 (1.2‐1.2)	<0.001	1.2 (1.2‐1.2)	<0.001
Race (White as ref.)								
Black	1.0 (1.0‐1.0)	0.874	1.0 (0.9‐1.0)	0.876	1.0 (0.9‐1.0)	0.019	1.0 (0.9‐1.0)	<0.001
Other	0.72 (0.70‐0.75)	<0.001	0.72 (0.69‐0.75)	<0.001	0.73 (0.70‐0.76)	<0.001	0.72 (0.69‐0.75)	<0.001
Marital (Married as ref.)	1.2 (1.2‐1.2)	<0.001	1.2 (1.1‐1.2)	<0.001	1.1 (1.1‐1.1)	<0.001	1.1 (1.1‐1.1)	<0.001
Histology (AD as ref.)								
SQC	1.3 (1.2‐1.3)	<0.001	1.3 (1.2‐1.3)	<0.001	1.1 (1.1‐1.2)	<0.001	1.1 (1.1‐1.2)	<0.001
Other	1.1 (1.0‐1.1)	0.006	1.1 (1.0‐1.1)	<0.001	1.0 (0.9‐1.1)	0.994	1.0 (0.9‐1.1)	0.725
LCC	1.4 (1.3‐1.5)	<0.001	1.4 (1.3‐1.5)	<0.001	1.3 (1.2‐1.4)	<0.001	1.3 (1.2‐1.4)	<0.001
Unspecified	1.4 (1.4‐1.4)	<0.001	1.4 (1.4‐1.4)	<0.001	1.2 (1.2‐1.2)	<0.001	1.2 (1.2‐1.2)	<0.001
Grade (1 as ref.)								
2	1.3 (1.2‐1.4)	<0.001	1.3 (1.2‐1.4)	<0.001	1.3 (1.2‐1.4)	<0.001	1.3 (1.2‐1.4)	<0.001
3	1.7 (1.5‐1.8)	<0.001	1.7 (1.6‐1.8)	<0.001	1.6 (1.5‐1.7)	<0.001	1.6 (1.5‐1.7)	<0.001
4	1.9 (1.7‐2.1)	<0.001	2.0 (1.8‐2.2)	<0.001	1.7 (1.5‐1.9)	<0.001	1.8 (1.6‐2.0)	<0.001
Unknown	1.6 (1.5‐1.8)	<0.001	1.7 (1.5‐1.8)	<0.001	1.5 (1.4‐1.6)	<0.001	1.5 (1.4‐1.7)	<0.001
Number of distant metastasis (0 as ref.)^b^								
1	1.1 (1.0‐1.1)	<0.001	1.1 (1.1‐1.1)	<0.001	1.1 (1.1‐1.1)	<0.001	1.2 (1.1‐1.2)	<0.001
2	1.3 (1.3‐1.4)	<0.001	1.4 (1.3‐1.4)	<0.001	1.5 (1.5‐1.6)	<0.001	1.6 (1.5‐1.6)	<0.001
3	1.6 (1.5‐1.6)	<0.001	1.6 (1.6‐1.7)	<0.001	1.9 (1.8‐1.9)	<0.001	1.9 (1.9‐2.0)	<0.001
4	1.5 (1.4‐1.7)	<0.001	1.6 (1.5‐1.8)	<0.001	1.8 (1.7‐2.1)	<0.001	2.0 (1.8‐2.2)	<0.001
Chemotherapy (No as ref.)	0.42 (0.41‐0.43)	<0.001	0.43 (0.42‐0.44)	<0.001	0.42 (0.41‐0.43)	<0.001	0.43 (0.42‐0.44)	<0.001
Surgery (No as ref.)	0.45 (0.42‐0.48)	<0.001	0.44 (0.41‐0.47)	<0.001	0.49 (0.45‐0.52)	<0.001	0.48 (0.45‐0.51)	<0.001
Radiotherapy (No as ref.)	0.87 (0.85‐0.89)	<0.001	0.89 (0.87‐0.91)	<0.001	0.89 (0.87‐0.90)	<0.001	0.90 (0.88‐0.92)	<0.001

n, number of cases/controls; OS, overall survival; CSS, cancer‐specific survival; PSM, propensity score matching; RT, radiotherapy; AD, adenocarcinoma; SQC, squamous cell carcinoma; Other, other specified carcinoma; LCC, large cell carcinoma; Unspecified, unspecified malignant neoplasm; HR, hazard ratio; CI, confidence interval.

a Derived from COX regression analysis.

All the variables selected in this table were included in the multivariate analysis.

b Number of distant metastasis included four sites of distant metastasis at diagnose SEER provided: bone, brain, liver, and lung.

Table 3 shows the OS and CSS of different metastases to the four selected metastatic sites, brain, bone, liver, and lung, at diagnosis with or without RT after PSM. PSM was performed for each subgroup including the variables of age, gender, race, marital status, histological type, grade, T stage, N stage, chemotherapy, surgery, and RT. RT was found to be associated with significantly improved survival of NSCLC patients with metastases to the brain (*P *<* *0.001) but not to the bone (*P *=* *0.134), lungs (*P = *0.055), or liver (*P *=* *0.399). Interestingly, patients who developed metastases at more than two sites, that is, patients with polymetastases, receiving RT had both better OS (*P *<* *0.001) and better CSS (*P = *0.012).

**Table 3:** OS and CSS of lung cancer with distant metastasis to bone, brain, liver, and lung with or without radiotherapy after PSM^a^

Metastatic site	n	OS (95% CI)^b^mo	*P* c	CSS (95% CI)^b^mo	*P* c
Brain^d^					
No RT	2253	5.6 (5.2‐6.0)	<0.001	6.3 (5.8‐6.9)	<0.001
RT	2253	7.5 (7.0‐8.0)		8.1 (7.6‐8.7)	
Bone^d^					
No RT	5515	8.2 (7.9‐8.6)	0.027	9.0 (8.6‐9.3)	0.134
RT	5515	8.2 (7.9‐8.5)		8.8 (8.4‐9.1)	
Liver^d^					
No RT	2113	6.5 (6.0‐7.0)	0.175	7.0 (6.5‐7.6)	0.399
RT	2113	6.4 (6.0‐6.8)		6.8 (6.4‐7.2)	
Lung^d^					
No RT	3479	9.4 (9.0‐9.9)	0.040	10.4 (9.9‐10.9)	0.055
RT	3479	10.0 (9.5‐10.4)		10.6 (10.1‐11.1)	
All sites^e^					
No RT	11 025	9.7 (9.5‐10.0)	0.328	10.7 (10.4‐11.0)	0.648
RT	11 025	9.5 (9.2‐9.7)		10.1 (9.9‐10.4)	
≥2 sites^f^					
No RT	3874	6.7 (6.3‐7.0)	<0.001	7.4 (7.0‐7.7)	0.012
RT	3874	7.1 (6.8‐7.4)		7.5 (7.2‐7.9)	

n, number of cases/controls; OS, overall survival; CSS, cancer‐specific survival; CI, confidence interval; PSM, propensity score matching; RT, radiotherapy.

a PSM were done in each subgroup using variables including: age, gender, race, marital, histological types, grade, T stage, N stage, chemotherapy, surgery, and radiotherapy.

b Derived from Kaplan‐Meier survival analysis.

c Derived from log‐rank test statistics.

d Bone, brain, liver, and lung are four sites of distant metastasis at diagnose SEER provided.

e All sites: With one or more metastatic sites in bone, brain, liver, and lung.

f ≥2 sites: With two or more metastatic sites in bone, brain, liver, and lung.

Table 4 further shows the OS and CSS for different histologies. PSM was performed in each subgroup including the variables of age, gender, race, marital status, grade, T stage, N stage, chemotherapy, surgery, RT, and metastatic sites of bone, brain, liver, and lung. RT was found to be associated with a significant improvement in survival among patients with a histology of SQC (*P *<* *0.001), LCC (*P = *0.044) or unspecified malignant neoplasms (*P *<* *0.001) but not among patients with a histology of AD (*P *=* *0.205) or other specified carcinomas (*P *=* *0.119). As a whole, for all stage IV NSCLC patients, RT was associated with improvements in neither OS (*P *=* *0.857) nor CSS (*P *=* *0.080).

**Table 4:** OS and CSS between different histology of cancer with or without radiotherapy after PSM^a^

Histology	Treatment	n	OS (95% CI)^b^ mo	*P* c	CSS (95% CI)^b^ mo	*P* c
AD	No RT	5429	11.6 (11.2‐12.0)	0.423	12.6 (12.1‐13.0)	0.205
	RT	5429	11.0 (10.6‐11.4)		11.8 (11.4‐12.2)	
SQC	No RT	2397	7.6 (7.1‐8.1)	<0.001	8.6 (8.0‐9.1)	<0.001
	RT	2397	9.6 (9.1‐10.1)		10.7 (10.1‐11.3)	
Other	No RT	448	9.8 (8.4‐11.2)	0.061	10.8 (9.3‐12.4)	0.119
	RT	448	10.6 (9.4‐11.8)		11.2 (9.9‐12.6)	
LCC	No RT	222	6.3 (5.0‐7.5)	0.041	6.6 (5.3‐7.9)	0.044
	RT	222	8.2 (6.6‐9.8)		8.6 (6.9‐10.3)	
Unspecified	No RT	1746	6.8 (6.3‐7.4)	<0.001	7.7 (7.0‐8.3)	<0.001
	RT	1746	8.9 (8.3‐9.5)		9.6 (8.9‐10.3)	
All NSCLC	No RT	14 066	10.8 (10.5‐11.0)	0.857	11.8 (11.6‐12.1)	0.080
	RT	14 066	10.4 (10.1‐10.6)		11.1 (10.9‐11.4)	

PSM, propensity score matching; n, number of cases/controls; RT, radiotherapy; OS, overall survival; CSS, cancer‐specific survival; CI, confidence interval; AD, adenocarcinoma; SQC, squamous cell carcinoma; Other, other specified carcinoma; LCC, large cell carcinoma; Unspecified, unspecified malignant neoplasm; NSCLC, non‐small cell lung cancer.

PSM were done in each subgroup using variables including: age, gender, race, marital, grade, T stage, N stage, chemotherapy, surgery, radiotherapy, and metastatic sites of bone, brain, liver, and lung.

Derived from Kaplan‐Meier survival analysis.

Derived from log‐rank test statistics.

Table 5 shows the interplay of metastatic sites with histology types in the treatment of RT for stage IV NSCLC. PSM was performed for each subgroup including the variables of age, gender, race, marital status, grade, T stage, N stage, chemotherapy, surgery, and RT. A statistical interaction was found among different pathological types. For SQC, RT was found to universally and significantly improve the survival of patients for almost all metastatic sites (*P *<* *0.001) in addition to patients without metastasis (*P *<* *0.001) most likely due to treatment of the primary site. For LCC and unspecified malignant neoplasms, there was a trend toward improving OS and CSS, although most of the analyses were not statistically significant, possibly due to the small sample size. For AD, radiation conferred a survival benefit only for patients with brain metastasis (OS: 8.7 months vs 6.8 months, *P *<* *0.001; CSS: 9.2 months vs 7.6 months, *P *<* *0.001). Interestingly, patients who developed metastases at more than two sites, that is, patients with polymetastases, receiving RT had both better OS (*P *<* *0.001) and better CSS (*P = *0.012). It is also intriguing that patients with polymetastases receiving RT tended to have both better OS and better CSS for all histological types except SQC.

**Table 5:** OS and CSS between different histology and metastatic sites of NSCLC with or without radiotherapy after PSM^a^

Histology	Treatment	n	OS (95%CI)^b^ mo	*P* c	CSS (95%CI)^b^ mo	*P* c
Brain metastasis^d^						
AD	No RT	1265	6.8 (6.1‐7.5)	<0.001	7.6 (6.9‐8.4)	<0.001
	RT	1265	8.7 (8.0‐9.4)		9.2 (8.5‐10.0)	
SQC	No RT	266	3.7 (2.9‐4.6)	0.037	4.3 (3.3‐5.3)	0.093
	RT	266	4.6 (3.7‐5.5)		4.9 (3.9‐5.9)	
Other	No RT	85	6.0 (3.4‐8.6)	0.139	6.2 (3.5‐8.9)	0.095
	RT	85	6.4 (4.7‐8.1)		7.0 (5.1‐8.9)	
LCC	No RT	42	4.2 (2.5‐5.9)	0.114	4.5 (2.7‐6.4)	0.128
	RT	42	7.4 (3.9‐11.0)		8.6 (4.3‐12.8)	
Unspecified	No RT	441	4.2 (3.4‐5.0)	0.001	4.8 (3.9‐5.8)	0.002
	RT	441	5.8 (4.8‐6.8)		6.4 (5.3‐7.5)	
Bone metastasis^d^						
AD	No RT	2927	10.1 (9.5‐10.6)	0.995	10.9 (10.3‐11.5)	0.747
	RT	2927	9.6 (9.2‐10.1)		10.3 (9.8‐10.8)	
SQC	No RT	807	5.2 (4.6‐5.8)	0.005	5.7 (5.1‐6.4)	0.015
	RT	807	6.0 (5.4‐6.6)		6.5 (5.8‐7.2)	
Other	No RT	184	7.8 (6.1‐9.5)	0.105	8.6 (6.7‐10.5)	0.233
	RT	184	9.1 (7.4‐10.7)		9.3 (7.6‐11.1)	
LCC	No RT	74	4.9 (3.3‐6.5)	0.364	5.3 (3.6 ‐7.0)	0.445
	RT	74	5.7 (3.9‐7.6)		6.1 (4.1‐8.1)	
Unspecified	No RT	761	6.0 (5.3‐6.8)	0.029	6.6 (5.8‐7.5)	0.089
	RT	761	6.6 (5.9‐7.4)		7.1 (6.2‐8.0)	
Liver metastasis^d^						
AD	No RT	852	7.5 (6.7‐8.3)	0.451	8.1 (7.2‐8.9)	0.249
	RT	852	6.7 (6.0‐7.3)		7.0 (6.3‐7.6)	
SQC	No RT	337	4.6 (3.9‐5.3)	0.013	5.0 (4.2‐5.9)	0.028
	RT	337	5.8 (4.9‐6.6)		6.1 (5.2‐7.1)	
Other	No RT	95	6.5 (4.3‐8.7)	0.092	6.9 (4.6‐9.2)	0.110
	RT	95	8.7 (6.4‐11.0)		9.1 (6.7‐11.4)	
LCC	No RT	32	3.0 (1.2‐4.7)	0.116	3.0 (1.2‐4.7)	0.116
	RT	32	5.0 (2.8‐7.1)		5.0 (2.8‐7.1)	
Unspecified	No RT	328	4.4 (3.6‐5.2)	0.146	4.7 (3.9‐5.6)	0.186
	RT	328	5.1 (4.2‐6.1)		5.5 (4.4‐6.6)	
Lung metastasis^d^						
AD	No RT	1596	11.3 (10.5‐12.0)	0.166	12.1 (11.4 ‐12.9)	0.113
	RT	1596	10.3 (9.6‐11.0)		11.0 (10.3‐11.8)	
SQC	No RT	696	8.4 (7.5‐9.3)	<0.001	9.1 (8.1‐10.1)	0.001
	RT	696	10.5 (9.5‐11.5)		11.4 (10.3‐12.5)	
Other	No RT	90	14.0 (10.1‐18.0)	0.104	14.8 (10.6‐18.9)	0.109
	RT	90	9.3 (6.5‐12.1)		9.7 (6.7‐12.6)	
LCC	No RT	41	4.6 (2.8‐6.4)	0.100	4.6 (2.8‐6.4)	0.100
	RT	41	7.2 (4.5‐9.9)		7.2 (4.5‐9.9)	
Unspecified	No RT	436	6.3 (5.4‐7.3)	0.008	7.1 (6.0‐8.2)	0.034
	RT	436	8.1 (6.9‐9.2)		8.5 (7.2‐9.7)	
Non metastasis^e^						
AD	No RT	976	15.2 (14.1‐16.3)	0.318	16.6 (15.4‐17.8)	0.361
	RT	976	15.9 (14.8‐17.0)		17.3 (16.1‐18.5)	
SQC	No RT	703	9.6 (8.9‐10.9)	<0.001	11.1 (10.0‐12.4)	<0.001
	RT	703	12.4 (11.3‐13.6)		14.2 (12.8‐15.6)	
Other	No RT	83	15.1 (10.7‐19.6)	0.340	17.3 (12.4‐22.2)	0.244
	RT	83	10.5 (7.9‐13.0)		11.2 (8.4‐14.0)	
LCC	No RT	41	6.7 (4.2‐9.3)	0.124	7.0 (4.3‐9.6)	0.063
	RT	41	10.8 (6.4‐15.1)		12.5 (7.2‐17.7)	
Unspecified	No RT	370	9.7 (8.4‐11.1)	0.132	11.1 (9.5‐12.6)	0.215
	RT	370	11.3 (9.8‐12.9)		12.5 (10.8‐14.3)	
≥2 sites^f^						
AD	No RT	2285	8.1 (7.6‐8.6)	0.088	8.9 (8.3‐9.4)	0.221
	RT	2285	8.4 (7.9‐8.9)		9.0 (8.5‐9.3)	
SQC	No RT	529	4.5 (3.8‐5.2)	0.248	4.9 (4.1‐5.7)	0.295
	RT	529	4.6 (4.0‐5.2)		4.9 (4.3‐5.6)	
Other	No RT	168	6.2 (4.7‐7.7)	0.057	6.5 (4.9‐8.1)	0.061
	RT	168	7.6 (6.1‐9.1)		7.9 (6.4‐9.4)	
LCC	No RT	70	2.8 (2.1‐3.5)	0.002	2.8 (2.1‐3.5)	0.003
	RT	70	5.4 (3.8‐6.9)		5.4 (3.8‐6.9)	
Unspecified	No RT	629	4.4 (3.7‐4.9)	0.003	4.8 (4.2‐5.5)	0.021
	RT	629	5.4 (4.7‐6.2)		5.7 (4.9‐6.5)	

n, number of cases/controls; OS, overall survival; PSM, propensity score matching; RT, radiotherapy; NSCLC, non‐small cell lung cancer; AD, adenocarcinoma; SQC, squamous cell carcinoma; Other, other specified carcinoma; LCC, large cell carcinoma; Unspecified, unspecified malignant neoplasm.

a PSM were done in each subgroup using variables including age, gender, race, marital, grade, T stage, N stage, chemotherapy, surgery, and radiotherapy.

b Derived from Kaplan‐Meier survival analysis.

c Derived from log‐rank test statistics.

d Bone, brain, liver and lung are four sites of distant metastasis at diagnose SEER provided.

e Non metastasis: With no metastatic sites in bone, brain, liver, and lung.

f ≥2 sites: With two or more metastatic sites in bone, brain, liver, and lung.

## 4 DISCUSSION

To our knowledge, the present study is the first population‐based analysis using PSM to assess the role of RT in treating metastatic NSCLC. In this study, after conducting both multivariate regression and PSM analyses, we found that RT can generally confer a survival benefit for most histological types of stage IV NSCLC, except AD. The beneficial effect on survival observed from the SEER database highlights the importance of RT in the management of stage IV NSCLC. The efficacy of systemic therapy in metastatic NSCLC has continuously, although slowly, improved. Even before the era of targeted therapy, select patients with stage IV NSCLC could achieve long‐term survival through aggressive, multimodality treatments including surgery and RT.^22^ The rapid advances in molecular targeted therapy and immunotherapy in the treatment of metastatic NSCLC have helped to greatly prevent or delay additional metastases.

Theoretically, local control of the primary and metastatic tumor can reduce the intra‐thoracic disease burden and symptoms^6^ and might result in a better OS.^7^, ^8^, ^9^, ^11^, ^22^, ^23^ This notion is supported by a study of extensive‐stage SCLC.^10^ It remains controversial whether RT can confer a survival benefit in stage IV NSCLC patients, especially for those with non‐oligometastatic disease. First, the survival benefits observed in these trials might be driven by the effects of RT on the oligometastatic disease. The term “oligometastatic” was first proposed by Hellman and Weichselbaum^24^ and describes metastases at 1‐5 sites. A disease spectrum might exist between the oligometastatic state and the extensive metastatic state. A select cohort of stage IV NSCLC patients apparently survived better than stage III patients, indicating that stage IV NSCLC might be heterogeneous in nature.^23^ Emerging evidence shows that local therapy could be potentially curative for oligometastasis.^25^, ^26^, ^27^, ^28^

A recent systematic review by Ashworth et al^29^ found that definitive treatment of the primary tumor was a good prognostic factor for survival of NSCLC patients with 1‐5 metastases. In addition, Ashworth et al^30^ performed an individual patient data meta‐analysis and found that long‐term survival is common in patients with metachronous oligometastases. Through another meta‐analysis, Li et al^7^ subsequently found that synchronous oligometastatic NSCLC patients might benefit from aggressive thoracic therapy. In fact, radical treatment of oligometastatic NSCLC might be equally effective for synchronous and metachronous manifestations.^13^ Thus, radical treatment of extensive metastatic NSCLC might also improve prognosis in select cases. For example, we found that radiation treatment in patients with two or more organ metastases, which most likely indicates extensive metastases,^31^ also leads to better OS and CSS.

Palliative TRT was also shown to be safe and might be beneficial for metastatic NSCLC patients with controlled extra‐thoracic diseases.^32^ However, one study found that the use of palliative TRT in stage IV NSCLC was associated with younger patients, the receipt of chemotherapy, and having undergone surgery of metastatic sites, which indicates that the survival benefits of palliative TRT may be due to confounding factors.^33^ Controlling the primary tumor was found to prolong patient survival and may lead to long‐term survival of patients with stage IV NSCLC.^34^, ^35^ Refusing palliative RT led to poor survival.^36^ Both the 2011 American Society for Radiation Oncology (ASTRO) Guideline and the 2014 European Society for Medical Oncology (ESMO) Guideline concluded that RT plays a major role in controlling the symptoms of metastases, such as painful chest wall disease, superior vena cava syndrome, soft tissue, or neural invasion. Therefore, there is no doubt that palliative TRT plays an important role in metastatic lung cancer. In addition, the ASTRO guideline noted that higher dose/fractionation RT regimens are associated with modest improvements in survival, suggesting that RT might have an additional role other than its palliative effects.^37^, ^38^ Aggressive palliative radiation doses delivered to the primary tumor were associated with better OS and local control in patients with stage IV NSCLC.^8^ Yun Chiang et al^32^ found that high thoracic RT doses (median dose of 55 Gy) resulted in greater survival. However, while high RT doses render higher tumor control rates, there is also a higher than normal probability of complications. Thus, the safety and efficacy of RT needs to be carefully considered. It is of paramount importance to identify the subgroup of patients with extensive metastasis who may be potentially cured by adding RT, after careful consideration of the safety and efficacy, to systemic therapy.

In the era of two‐dimensional RT (2D‐RT), TRT has long been used only for palliative care in metastatic NSCLC.^8^, ^39^ New RT techniques, such as stereotactic body radiotherapy (SBRT), have reduced radiation doses to normal tissues and increased the dose to the tumor.^9^, ^40^ Simultaneous RT to >5 metastatic sites has been shown to be feasible, tolerable and effective. For example, RT using γ knife surgery has been widely used in patients with >10 brain metastases.^41^ A current phase II randomized trial (NCT Identifier: NCT02045446)^42^ is enrolling patients to receive either consolidative SBRT plus maintenance chemotherapy or maintenance chemotherapy alone for limited metastatic NSCLC. Accrual was ended early after an unplanned interim analysis found a significant improvement in progression‐free survival (PFS), from 3.5 to 9.7 months, with the addition of SBRT. Toxicity was similar in each arm. This study, together with previous data, has led to the current phase III trial (Identifier: NRG‐LU 002), which is evaluating the effect of SBRT in addition to maintenance chemotherapy on the OS of limited metastatic NSCLC patients. Moreover, as reported by Rusthoven et al,^43^ the predominant pattern of failure in advanced NSCLC after first‐line systemic therapy is local recurrence, which justifies adding SBRT treatment to improve the time to disease progression. Barton underlined the importance of designing this study to reflect real‐world treatment approaches, which will make clinicians and patients more comfortable with the approach of consolidative local therapy.^44^ Moreover, because of the remarkable physical and biophysical advantages, more advanced radiation techniques, such as proton^45^ and heavy ion therapy,^46^ could be developed for treating stage IV NSCLC.^37^, ^38^ Therefore, with the development of new radiation technologies, more metastatic sites can be effectively treated by RT with tolerable toxicity. Furthermore, the approach of consolidative local therapy will likely become more relevant as systemic therapies, such as immunotherapies, improve.^44^

Nearly all previous studies of NSCLC patients with oligometastases were nonrandomized and had small sample sizes. To date, the only randomized phase III trial was limited to brain metastases and showed no clinical benefits of local stereotactic radiosurgery.^47^ However, that trial was underpowered because of a small sample size and was terminated early due to slow recruitment because was approved in 2012 in Korea as the first‐line treatment for patients with EGFR mutations.^47^ Additionally, more than 84% of the included patients had AD. The subsequent switch to EGFR TKIs would significantly change the observed outcome. In this sense, our study may be more indicative of the real‐world situation of AD patients in a post‐EGFR TKI era.

Consistent with the results of previous research, we found that the influence of histological differences on prognosis might be greater than that of metastatic site differences.^48^ SQC, LCC, and unspecified malignant neoplasm were found to be sensitive to RT regardless of metastatic sites, whereas ADs seemed less responsive to RT, except in cases of brain metastasis. Although selection bias could not be ruled out as the cause of the differences among affected organs,^31^ the utilization of PSM in the current study effectively reduced this possibility. Unsurprisingly, we found that AD patients had better survival than patients with other histological types for all metastatic sites regardless of therapy. Thus, this survival benefit may not be attributable to RT but rather to targeted therapy, namely EGFR TKI therapy, since we recruited newly diagnosed NSCLC patients from 2010 to 2012. During this timeframe, EGFR TKI therapy was the standard of care for metastatic lung AD. It should be noted that the use of EGFR TKIs, when appropriate, significantly prolonged the post‐recurrence/metastatic survival of AD patients. Therefore, the survival benefit of applying RT might be effectively masked in this population. In this sense, our study may be more indicative of the real‐world situation of AD patients in a post‐EGFR TKI era.

Unlike RCTs, the SEER database usually has high completeness and is representative of the real‐world patient population. RCTs are prone to selection bias due to the recruitment of a specific group of patients of interest, thus limiting the generalizability of the findings. Trials usually set age limits or select patient groups with a favorable outcome. For example, the European Organization for Research and Treatment of Cancer (EORTC) study used an inclusive age limit of 75 years.^49^ We did not set an age limit to the study cohort, and we did not refine the results to include chemotherapy responders, many of whom might experience progression after chemotherapy, making the current findings more applicable to real‐world settings.

We acknowledge several limitations of this study. First, as with any observational study, the possibility of bias is a concern. We used the PSM method, which might reduce the bias caused by the imbalanced distributions of the measured covariates. However, bias from unmeasured factors is unavoidable. Second, although our results might be applicable to real‐world settings, we acknowledge that differences in the radiation dose timing, intent, methods, side effects, and second‐line chemotherapy may all have contributed to study bias. However, RT in the SEER database is defined as using RT during the first course of cancer‐directed therapy, with no information on the dose or intended target. Third, the SEER database provides no information on chemotherapy regimens, which might be significantly correlated with the survival of advanced NSCLC patients. However, since systematic therapy rather than RT is the standard of care for stage IV NSCLC, most of this cohort of patients received systematic therapy. We adjusted chemotherapy as a covariable in all the multivariate analyses. In addition, as suggested by the National Comprehensive Cancer Network (NCCN) guidelines and other guidelines, chemotherapy regimens for patients with stage IV NSCLC are usually platinum‐based duplexes as first‐line therapy. There are no appreciable differences in OS correlated with the selection of platinum‐based duplexes for advanced NSCLC patients. Thus, the selection of chemotherapy regimens should not differentially affect the survival between NSCLC patients who received RT and those did not. Forth, metastatic sites other than bone, brain, lung, and liver are not coded in the SEER database, which might lead to underestimation of the number of metastatic sites. Although more details would be beneficial, we aimed to show the general survival advantage of RT for stage IV NSCLC patients. In other words, our study was conducted from a qualitative, rather than quantitative, perspective. In this regard, we believe that the currently available data in the SEER database serve this aim very well. In the current analysis, we did not aim to define the type, timing, dosage, intent, or methods of RT that should be used in stage IV NSCLC. In addition, the SEER database does not provide data on the risk factors of NSCLC, such as smoking, which may have influenced survival. Nevertheless, the study participants were recruited through a representative national database, thus reducing possible selection biases. When we changed the cutoffs of some variables, such as age, the OS and CSS results did not markedly change and, thus, seem stable and valid.

This study shows that RT of the primary site and metastatic sites may significantly improve the survival of stage IV NSCLC patients. Further advancements in RT techniques may reduce its toxicity to normal tissues and allow increased doses to tumors, which may further improve the efficacy of the treatment.

## 5 CONCLUSION

The present study based on the large SEER database supports that radiation therapy in addition to chemotherapy might be beneficial for the survival of patients with metastatic NSCLC. Although well‐designed phase III RCTs are warranted to ascertain the value of radiotherapy in this setting, it is prudent to routinely select suitable patients for radiation therapy to the primary and metastatic sites in metastatic NSCLC based on chemotherapy.

## AVAILABILITY OF DATA AND MATERIALS

The datasets generated and/or analyzed during the current study are available in the SEER repository, [https://seer.cancer.gov/].

## CONFLICT OF INTEREST

Authors declare no conflicts of interest for this article.

## References

[1] Schiller JH , Harrington D , Belani CP , et al. Comparison of four chemotherapy regimens for advanced non‐small‐cell lung cancer. N Engl J Med. 2002;346(2):92‐98. 11784875 10.1056/NEJMoa011954

[2] Cohen MH , Williams GA , Sridhara R , et al. United States food and drug administration drug approval summary: Gefitinib (ZD1839; Iressa) tablets. Clin Cancer Res. 2004;10(4):1212‐1218. 14977817 10.1158/1078-0432.ccr-03-0564

[3] Thomas A , Liu SV , Subramaniam DS , Giaccone G . Refining the treatment of NSCLC according to histological and molecular subtypes. Nat Rev Clin Oncol. 2015;12(9):511‐526. 25963091 10.1038/nrclinonc.2015.90

[4] He J , Hu Y , Hu M , Li B . Development of PD‐1/PD‐L1 Pathway in Tumor Immune Microenvironment and Treatment for Non‐Small Cell Lung Cancer. Sci Rep. 2015;5(1):13110. 26279307 10.1038/srep13110 PMC4538573

[5] Morgensztern D , Waqar S , Subramanian J , Gao F , Govindan R . Improving survival for stage IV non‐small cell lung cancer: a surveillance, epidemiology, and end results survey from 1990 to 2005. J Thorac Oncol. 2009;4(12):1524‐1529. 19752759 10.1097/JTO.0b013e3181ba3634

[6] Rodrigues G , Macbeth F , Burmeister B , et al. International practice survey on palliative lung radiotherapy: Third International Consensus Workshop on Palliative Radiotherapy and Symptom Control. Clin Lung Cancer. 2012;13(3):225‐235. 22169482 10.1016/j.cllc.2011.11.002

[7] Li D , Zhu X , Wang H , Qiu M , Li N . Should aggressive thoracic therapy be performed in patients with synchronous oligometastatic non‐small cell lung cancer? A meta‐analysis J Thorac Dis. 2017;9(2):310‐317. 28275479 10.21037/jtd.2017.02.21 PMC5334109

[8] Fairchild A , Harris K , Barnes E , et al. Palliative thoracic radiotherapy for lung cancer: a systematic review. J Clin Oncol. 2008;26(24):4001‐4011. 18711191 10.1200/JCO.2007.15.3312

[9] Sun B , Brooks ED , Komaki RU , et al. 7‐year follow‐up after stereotactic ablative radiotherapy for patients with stage I non–small cell lung cancer: Results of a phase 2 clinical trial. Cancer. 2017;123(16):3031‐3039. 28346656 10.1002/cncr.30693 PMC5544582

[10] Slotman BJ , van Tinteren H , Praag JO , et al. Use of thoracic radiotherapy for extensive stage small‐cell lung cancer: a phase 3 randomised controlled trial. Lancet. 2015;385(9962):36‐42. 25230595 10.1016/S0140-6736(14)61085-0

[11] Lopez Guerra JL , Gomez D , Zhuang Y , et al. Prognostic impact of radiation therapy to the primary tumor in patients with non‐small cell lung cancer and oligometastasis at diagnosis. Int J Radiat Oncol Biol Phys. 2012;84(1):e61‐e67. 22503522 10.1016/j.ijrobp.2012.02.054 PMC3919541

[12] Arrieta O , Villarreal‐Garza C , Zamora J , et al. Long‐term survival in patients with non‐small cell lung cancer and synchronous brain metastasis treated with whole‐brain radiotherapy and thoracic chemoradiation. Radiat Oncol. 2011;6(1):166. 22118497 10.1186/1748-717X-6-166 PMC3235073

[13] Fleckenstein J , Petroff A , Schäfers H‐J , Wehler T , Schöpe J , Rübe C . Long‐term outcomes in radically treated synchronous vs. metachronous oligometastatic non‐small‐cell lung cancer. BMC Cancer. 2016;16(1):348. 27255302 10.1186/s12885-016-2379-x PMC4890277

[14] Mehta N , Mauer AM , Hellman S , et al. Analysis of further disease progression in metastatic non‐small cell lung cancer: implications for locoregional treatment. Int J Oncol. 2004;25(6):1677‐1683. 15547705

[15] Ahmad SS , Duke S , Jena R , Williams MV , Burnet NG . Advances in radiotherapy. Br Med J. 2012;345:e7765. 23212681 10.1136/bmj.e7765

[16] Hankey BF , Ries LA , Edwards BK . The surveillance, epidemiology, and end results program: a national resource. Cancer Epidemiol Biomarkers Prev. 1999;8:1117‐1121. 10613347

[17] Denise RL , David P , Check BS , et al. US lung cancer trends by histologic type. Cancer. 2011;193(1):118‐125.

[18] Little RJ , Rubin DB . Causal effects in clinical and epidemiological studies via potential outcomes: concepts and analytical approaches. Annu Rev Public Health. 2000;21:121‐145. 10884949 10.1146/annurev.publhealth.21.1.121

[19] Rosenbaum PR , Rubin DB . The central role of the propensity score in observational studies for causal effects. Biometrika. 1983;70(1):41‐55.

[20] Propensity score matching in SPSS. arXiv:1201.6385.

[21] World Health Organisation . Definition of an older or elderly person. WHO, Geneva: Switzerland, 2010 http://www.who.int/healthinfo/survey/ageingdefnolder/en/index.html. Accessed May 8, 2018.

[22] Hirashima T , Suzuki H , Okamoto N , et al. Important factors for achieving survival of five years or more in non‐small cell lung cancer patients with distant metastasis. Oncol Lett. 2014;8(1):327‐334. 24959271 10.3892/ol.2014.2107 PMC4063572

[23] Cheruvu P , Metcalfe SK , Metcalfe J , Chen Y , Okunieff P , Milano MT . Comparison of outcomes in patients with stage III versus limited stage IV non‐small cell lung cancer. Radiat Oncol. 2011;6(1):80. 21718501 10.1186/1748-717X-6-80 PMC3141527

[24] Hellman S , Weichselbaum RR . Oligometastases. J Clin Oncol. 1995;13(1):8‐10. 7799047 10.1200/JCO.1995.13.1.8

[25] Hasselle MD , Haraf DJ , Rusthoven KE , et al. Hypofractionated image‐guided radiation therapy for patients with limited volume metastatic non‐small cell lung cancer. J Thorac Oncol. 2012;7(2):376‐381. 22198429 10.1097/JTO.0b013e31824166a5

[26] Rusthoven CG , Yeh N , Gaspar LE . Radiation therapy for oligometastatic non‐small cell lung cancer ‐ theory and practice. Cancer J. 2015;21(5):404‐412. 26389766 10.1097/PPO.0000000000000143

[27] Bansal P , Rusthoven C , Boumber Y , Gan GN . The role of local ablative therapy in oligometastatic non‐small‐cell lung cancer: hype or hope. Futur Oncol. 2016;12(23):2713‐2727. 10.2217/fon-2016-0219 27467543

[28] Gomez DR , Blumenschein GR , Lee JJ , et al. Local consolidative therapy versus maintenance therapy or observation for patients with oligometastatic non‐small‐cell lung cancer without progression after first‐line systemic therapy: a multicentre, randomised, controlled, phase 2 study. Lancet Oncol. 2016;17(12):1672‐1682. 27789196 10.1016/S1470-2045(16)30532-0 PMC5143183

[29] Ashworth A , Rodrigues G , Boldt G , Palma D . Is there an oligometastatic state in non‐small cell lung cancer? A systematic review of the literature. Lung Cancer. 2013;82(2):197‐203. 24051084 10.1016/j.lungcan.2013.07.026

[30] Ashworth AB , Senan S , Palma DA , et al. An individual patient data metaanalysis of outcomes and prognostic factors after treatment of oligometastatic non‐small‐cell lung cancer. Clin Lung Cancer. 2014;15(5):346‐355. 24894943 10.1016/j.cllc.2014.04.003

[31] Su S , Hu Y , Ouyang W , et al. Might radiation therapy in addition to chemotherapy improve overall survival of patients with non‐oligometastatic Stage IV non‐small cell lung cancer?: secondary analysis of two prospective studies. BMC Cancer. 2016;16(1):908. 27871270 10.1186/s12885-016-2952-3 PMC5117544

[32] Chiang Y , Yang JCH , Hsu FM , et al. The response, outcome and toxicity of aggressive palliative thoracic radiotherapy for metastatic non‐small cell lung cancer patients with controlled extrathoracic diseases. PLoS ONE. 2015;10(12):e0145936. 26720170 10.1371/journal.pone.0145936 PMC4697816

[33] Chen AB , Cronin A , Weeks JC , et al. Palliative radiation therapy practice in patients with metastatic non‐small‐cell lung cancer: a Cancer Care Outcomes Research and Surveillance Consortium (CanCORS) Study. J Clin Oncol. 2013;31:558‐564. 23295799 10.1200/JCO.2012.43.7954 PMC3565181

[34] Rastogi M , Revannasiddaiah S , Gupta MK , et al. When palliative treatment achieves more than palliation: instances of long‐term survival after palliative radiotherapy. Indian J Palliat Care. 2012;18:117‐121. 23093827 10.4103/0973-1075.100829 PMC3477364

[35] Yamaguchi S , Ohguri T , Matsuki Y , et al. Palliative radiotherapy in patients with a poor performance status: the palliative effect is correlated with prolongation of the survival time. Radiat Oncol. 2013;8:166. 23829540 10.1186/1748-717X-8-166 PMC3707862

[36] Stavas MJ , Arneson KO , Ning MS , et al. The refusal of palliative radiation in metastatic non‐small cell lung cancer and its prognostic implications. J Pain Symptom Manage. 2015;49:1081‐1087. e1084. 25596010 10.1016/j.jpainsymman.2014.11.298

[37] Rodrigues G , Videtic GM , Sur R , et al. Palliative thoracic radiotherapy in lung cancer: an American Society for Radiation Oncology evidence‐based clinical practice guideline. Pract Radiat Oncol. 2011;1:60‐71. 25740118 10.1016/j.prro.2011.01.005 PMC3808743

[38] Reck M , Popat S , Reinmuth N , et al. Metastatic non‐small‐cell lung cancer (NSCLC): ESMO Clinical Practice Guidelines for diagnosis, treatment and follow‐up. Ann Oncol. 2014;25(Suppl 3):iii27‐iii39. 25115305 10.1093/annonc/mdu199

[39] Rodrigues G , Videtic GMM , Sur R , et al. Palliative thoracic radiotherapy in lung cancer: an American Society for Radiation Oncology evidence‐based clinical practice guideline. Pract Radiat Oncol. 2011;1(2):60‐71. 25740118 10.1016/j.prro.2011.01.005 PMC3808743

[40] Basler L , Kroeze SGC , Guckenberger M . SBRT for oligoprogressive oncogene addicted NSCLC. Lung Cancer. 2017;106:50‐57. 28285694 10.1016/j.lungcan.2017.02.007

[41] Lee C‐K , Lee SR , Cho JM , Yang KA , Kim S‐H . Therapeutic effect of gamma knife radiosurgery for multiple brain metastases. J KOREAN Neurosurg Soc. 2011;50(3):179‐184. 22102945 10.3340/jkns.2011.50.3.179 PMC3218174

[42] Iyengar P , Wardak Z , Gerber DE , et al. Consolidative radiotherapy for limited metastatic non–small‐cell lung cancer. JAMA Oncol. 2017;4:e173501. 10.1001/jamaoncol.2017.3501 PMC5833648 28973074

[43] Rusthoven KE , Hammerman SF , Kavanagh BD , Birtwhistle MJ , Stares M , Camidge DR . Is there a role for consolidative stereotactic body radiation therapy following first‐line systemic therapy for metastatic lung cancer? A patterns‐of‐failure analysis Acta Oncol. 2009;48(4):578‐583. 19373699 10.1080/02841860802662722

[44] Barton MK . Local consolidative therapy may be beneficial in patients with oligometastatic non‐small cell lung cancer. CA Cancer J Clin. 2017;67(2):89‐90. 28094844 10.3322/caac.21363

[45] Chang JY , Zhang X , Knopf A , et al. Consensus guidelines for implementing pencil‐beam scanning proton therapy for thoracic malignancies on behalf of the PTCOG thoracic and lymphoma subcommittee. Int J Radiat Oncol Biol Phys. 2017;99(1):41‐50. 28816159 10.1016/j.ijrobp.2017.05.014

[46] Durante M , Orecchia R , Loeffler JS . Charged‐particle therapy in cancer: clinical uses and future perspectives. Nat Rev Clin Oncol. 2017;14(8):483‐495. 28290489 10.1038/nrclinonc.2017.30

[47] Lim SH , Lee JY , Lee MY , et al. A randomized phase III trial of stereotactic radiosurgery (SRS) versus observation for patients with asymptomatic cerebral oligo‐metastases in non‐small‐cell lung cancer. Ann Oncol. 2015;26(4):762‐768. 25538174 10.1093/annonc/mdu584

[48] Zimmermann S , Dziadziuszko R , Peters S . Indications and limitations of chemotherapy and targeted agents in non‐small cell lung cancer brain metastases. Cancer Treat Rev. 2014;40(6):716‐722. 24759599 10.1016/j.ctrv.2014.03.005

[49] Slotman B , Faivre‐Finn C , Kramer G , et al. Prophylactic cranial irradiation in extensive small‐cell lung cancer. N Engl J Med. 2007;357(7):664‐672. 17699816 10.1056/NEJMoa071780

